# Accumulation of acetaldehyde in *aldh2.1*^*−/−*^ zebrafish causes increased retinal angiogenesis and impaired glucose metabolism

**DOI:** 10.1016/j.redox.2022.102249

**Published:** 2022-01-26

**Authors:** David Philipp Wohlfart, Bowen Lou, Chiara Simone Middel, Jakob Morgenstern, Thomas Fleming, Carsten Sticht, Ingrid Hausser, Rüdiger Hell, Hans-Peter Hammes, Julia Szendrödi, Peter Paul Nawroth, Jens Kroll

**Affiliations:** a Department of Vascular Biology and Tumor Angiogenesis, European Center for Angioscience (ECAS), Medical Faculty Mannheim, Heidelberg University, Mannheim, 68167, Germany; b Department of Internal Medicine I and Clinical Chemistry, Heidelberg University Hospital, Heidelberg, 69120, Germany; c NGS Core Facility, Medical Faculty Mannheim, Heidelberg University, Mannheim, 68167, Germany; d Institute of Pathology IPH, EM Lab, Heidelberg University Hospital, Heidelberg, 69120, Germany; e Metabolomics Core Technology Platform, Centre for Organismal Studies, Heidelberg University, Heidelberg, 69120, Germany; f Fifth Medical Department and European Center for Angioscience, Medical Faculty Mannheim, Heidelberg University, 68167, Mannheim, Germany

**Keywords:** RCS, reactive carbonyl species, ROS, reactive oxygen species, AGEs, advanced glycation endproducts, Glo, glyoxalase, Akr, aldo-keto reductase, Aldh, aldehyde dehydrogenase, AA, acetaldehyde, 4-HNE, 4-hydroxynonenal, MG, methylglyoxal, MDA, malondialdehyde, ACR, acrolein, JNK, C-Jun N-terminal kinases, MAPK, mitogen-activated protein kinases, *pdx1*, pancreatic and duodenal homeobox 1, VEGF, vascular endothelial growth factor, *gck*, glucokinase, *g6pc*, glucose-6-phosphatase, Aldehyde dehydrogenase (ALDH), Acetaldehyde (AA), Reactive carbonyl species (RCS), Glucose metabolism, Microvascular organ complications, Zebrafish

## Abstract

Reactive carbonyl species (RCS) are spontaneously formed in the metabolism and modify and impair the function of DNA, proteins and lipids leading to several organ complications. In zebrafish, knockout of the RCS detoxifying enzymes glyoxalase 1 (Glo 1), aldehyde dehydrogenase 3a1 (Aldh3a1) and aldo-ketoreductase 1a1a (Akr1a1a) showed a signature of elevated RCS which specifically regulated glucose metabolism, hyperglycemia and diabetic organ damage. *aldh2.1* was compensatory upregulated in *glo1*^*−/−*^ animals and therefore this study aimed to investigate the detoxification ability for RCS by Aldh2.1 in zebrafish independent of ethanol exposure. *aldh2.1* knockout zebrafish were generated using CRISPR/Cas9 and subsequently analyzed on a histological, metabolomic and transcriptomic level. *aldh2.1*^*−/−*^ zebrafish displayed increased endogenous acetaldehyde (AA) inducing an increased angiogenesis in retinal vasculature. Expression and pharmacological interventional studies identified an imbalance of *c*-Jun N-terminal kinase (JNK) and p38 MAPK induced by AA, which mediate an activation of angiogenesis. Moreover, increased AA in *aldh2.1*^*−/−*^ zebrafish did not induce hyperglycemia, instead AA inhibited the expression of glucokinase (*gck)* and glucose-6-phosphatase (*g6pc)*, which led to an impaired glucose metabolism. In conclusion, the data have identified AA as the preferred substrate for Aldh2.1's detoxification ability, which subsequently causes microvascular organ damage and impaired glucose metabolism.

## Highlights

• ALDH2.1 was compensatory upregulated in glyoxalase 1 zebrafish mutants.
• Loss of ALDH2.1 increases acetaldehyde leading to vascular retinal alterations.
• Acetaldehyde controls glucose metabolism via glucose-6-phosphate and glucokinase.
• Altered JNK and p38 cause microvascular complications.

## Graphical abstract

**Figure:**
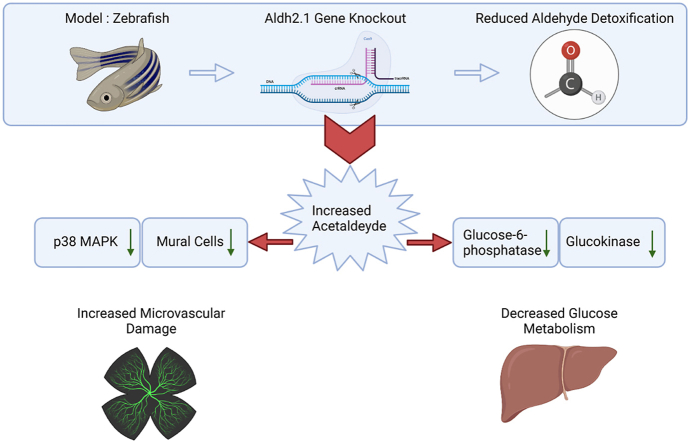

## 1 Introduction

Reactive carbonyl species (RCS) are a class of metabolites that show correlations to impaired glucose metabolism, insulin resistance and microvascular damage [[1], [2], [3], [4]]. The best studied representative of these molecules, methylglyoxal (MG), is known for its toxic capabilities as a precursor of advanced glycation end products (AGEs). Detoxification of MG is utilized by the glyoxalase (Glo) enzyme system consisting of *glo1* and *glo 2* [[5], [6], [7]]; [[5], [6], [7]] [[5], [6], [7]] however, recent studies in zebrafish and mice revealed that knockout of *glo1* only led to 50% increase of endogenous MG. Consequently, *glo1* knockout zebrafish, although showing an impaired glucose tolerance, did not develop organ damages and only after high calorie intake, they showed an altered retinal blood vasculature consistent with pathological findings in clinical diabetic retinopathy [8,9]. These results suggested that loss of the MG detoxifying ability of *glo1* can be compensated in vivo by other enzyme systems. In fact, activity measurements in *glo1* mutants for aldehyde dehydrogenases (Aldh) and aldo-keto reductases (Akr) indicated that these two enzyme families may act as alternative detoxification systems for MG [8,9]. Subsequent studies on different Aldh and Akr subclass members in zebrafish, including generation and analysis of *aldh3a1* and *akr1a1a* zebrafish mutants, found not MG, but 4-hydroxynonenal (4-HNE) for Aldh3a1 and acrolein (ACR) for Akr1a1a as the preferred detoxified RCS. Intriguingly, although glucose metabolism was impaired in both mutants, increased 4-HNE in *aldh3a1* mutants disrupted the pancreas leading to hyperglycemia and retinal vessel alterations, while increased ACR in *akr1a1a* mutants led to insulin resistance and hallmarks of diabetic retinopathy and diabetic nephropathy in adult animals [10,11]. In conclusion, the data have identified a specific signature of RCS and their corresponding detoxifying enzymes in regulating glucose metabolism and alterations in these systems led to impaired glucose tolerance, insulin resistance, hyperglycemia and microvascular complications.

*aldh2.1* expression was also increased in *glo1*^*−/−*^ zebrafish mutants [8]. *aldh2.1* is the zebrafish homolog to human *aldh2,* which can detoxify a variety of reactive metabolites, including acetaldehyde (AA), 4-HNE, malondialdehyde (MDA) and MG [[12], [13], [14], [15]]. Because Aldh2 oxidizes AA to acetic acid, it is well studied for its importance in alcohol metabolism and alcohol-induced stress complications, where it plays a critical role in alcoholic liver disease and cardiovascular disease [[16], [17], [18]]. In addition, loss of a*ldh2* can increase reactive oxygen species (ROS), which leads to mitochondria dysfunction and upregulation of cytochrome P450 2E1 (CYP2E1) [[19], [20], [21]]. Yet, Aldh2's contribution to reactive metabolite detoxification, regulation of glucose metabolism and formation of diabetic microvascular complications independently of ethanol exposure is not understood.

Therefore, the study aimed to evaluate the detoxification ability of Aldh2.1 for different RCS in zebrafish and to identify a potential regulatory function of Aldh2.1 on glucose metabolism, organ physiology and diseases. Our data identified that the loss of *aldh2.1* in zebrafish caused an increased endogenous AA concentration, which subsequently impaired the glucose metabolism and induced microvascular damages in retinal blood vessels in zebrafish larvae and adults.

## 2 Results

### 2.1 Generation and validation of aldh2.1^−/−^ knockout zebrafish

Recent studies in vertebrates and mammals indicated a major role of Aldh enzymes in detoxifying short and long chained RCS [12,13,15,22,23]. To investigate the impact of Aldh2.1 on RCS detoxification and regulation of glucose metabolism, we created an *aldh2.1* knockout zebrafish model as previous studies [8,24] suggested a compensatory function of *aldh2.1* in reactive metabolite detoxification.

**Fig. 1:**
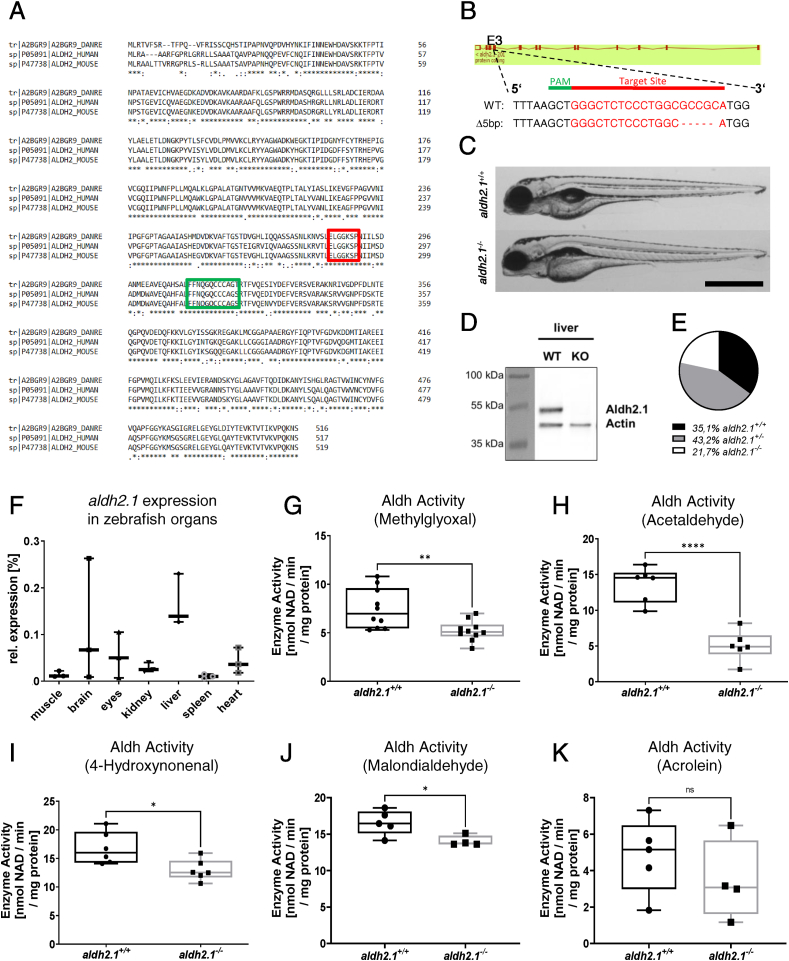
**Generation and validation of an *aldh2.1***^***−/−***^**zebrafish line. A**: Amino acid alignment of Aldh2 across different species displays a high similarity between zebrafish (first line), human (second line) and mouse (third line); glutamic acid active site (red) and cysteine active site (green) are indicated. **B**: *aldh2.1*-CRISPR-target site was designed for exon 3 and CRISPR/Cas9 induced five nucleotides base pair deletion was selected for further studies. **C**: Images of *aldh2.1*^*+/+*^ and *aldh2.1*^*−/−*^ larvae showed no difference at 5 dpf. Black scale bar: 500 μm. **D**: Western blot for Aldh2.1 and Actin proteins in adult liver confirmed the loss of Aldh2.1 in *aldh2.1*^*−/−*^ mutants. **E**: Adult a*ldh2.1*^*+/−*^ and *aldh2.1*^*−/−*^ animals are underrepresented according to the Mendelian distribution. a*ldh2.1*^*+/+*^ = 99 *(35.1%),* a*ldh2.1*^*+/−*^ = 122 (43.2%) and *aldh2.1*^*−/−*^ = 61 (21.7%). **F**: *aldh2.*1 mRNA expression in *aldh2.1*^*+/+*^ zebrafish was highest in liver (15%) followed by brain (6.8%) and eyes (5%). Expression was quantified via RT-qPCR and normalized to b2m, n = 3, one organ per sample. **G-K**: *aldh2.1*^*−/−*^ mutants displayed decreased Aldh enzyme activity with MG (**G**) as substrate, n = 10; AA (**H**) as substrate, n = 6; 4-HNE (**I**) as substrate, n = 6 and MDA (**J**) as substrate, n = 4–5, but unaltered Aldh enzyme activity with ACR (**K**) as substrate, n = 4–5. Enzyme activity was quantified via spectrophotometry of NAD metabolic rate (nmol NAD \ min \ mg protein) in zebrafish lysates at 96 hpf, 50 larvae per clutch. Statistical analysis was done via Student's t-test, ns = not significant, *p < 0.05, **p < 0.01, ***p < 0.001, ****p < 0.0001. (For interpretation of the references to colour in this figure legend, the reader is referred to the Web version of this article.)

The Aldh2.1 enzyme is expressed in human, mouse and zebrafish. However, similarities of the enzyme amino acid sequence between different species has not been described yet. Therefore, the first step of the study was the alignment of amino acid sequences between zebrafish and human, and zebrafish and mouse resulting in a 78.2% similarity between zebrafish and human and likewise 77.4% for zebrafish and mouse. The cysteine and glutamic amino acids in the active sites across the species are completely preserved and stay unchanged throughout each species (Fig. 1A). Up to date, an *aldh2.1*^*−/−*^ zebrafish line has not been generated, thus a knockout for *aldh2.*1 by using CRISPR/Cas9 technology was established. First, CRISPR-guideRNA (gRNA) targeting exon 3 of *aldh2.1* was synthesized and injected together with Cas9 mRNA into one-cell stage *Tg(fli1:EGPF)* zebrafish embryos. Sequencing identified a reading frame shift mutant due to a deletion of five nucleotides, which was then utilized for further breeding and studies (Fig. 1B). To validate successful generation of the homozygous *aldh2.1*^*−/−*^ mutant, a Western blot was performed and showed the complete loss of the Aldh2.1 protein in liver (Fig. 1D). The gross morphology of *aldh2.1*^*−/−*^ larvae at 5 day-post-fertilization (dpf) was not altered compared to *aldh2.1*^*+/+*^ littermates (Fig. 1C); however, in few *aldh2.1*^*−/−*^ embryos/larvae the livers appeared enlarged. Intriguingly, survival rates of adult *aldh2.1*^*−/−*^ animals deviated from the estimated Mendelian distribution and was significantly lower than expected. Out of 282 adult zebrafish, 99 (35.1%) were *aldh2.1*^*+/+*^, 122 (43.2%) were *aldh2.1*^*+/−*^ and only 61 (21.7%) had the Δ5 bp version *aldh2.1*^*−/−*^ (Fig. 1E), These results showed that permanent loss of Aldh2.1 negatively affects the survival of zebrafish.

Since expression of *aldh2.1* in adult zebrafish was unknown, RT-qPCR analysis was used to study expression of *aldh2.1* throughout zebrafish organs (Fig. 1F). We found highest expression of *aldh2.1* in liver (15%) followed by brain (6.8%) and eyes (5%) as compared to the expression of the house keeping gene b2m. Overall, these results confirmed expression of *aldh2.1* in zebrafish organs comparable to other vertebrates [25]. Next, a set of Aldh enzyme activity measurements were performed using different RCS as substrates to confirm that the *aldh2.1* knockout resulted in a functional reduction of total Aldh enzyme activity. A significant reduction of total Aldh enzyme activity in lysates from *aldh2.1*^*−/−*^ larvae could be observed with the following substrates: AA (67%, Fig. 1H), MG (31%, Fig. 1G), 4-HNE (23%, Fig. 1I) and MDA (16%, Fig. 1J). Total Aldh enzyme activity with ACR (Fig. 1K) as substrate was also reduced by 28%, but was not significant. These results not only further demonstrated the successful generation of *aldh2.1*^*−/−*^ mutants, but also identified a capacity of the Aldh2.1 enzyme in the detoxification of several RCS in zebrafish.

### 2.2 Loss of aldh2.1 led to angiogenesis in the retinal vasculature of zebrafish larvae and adults

**Fig. 2:**
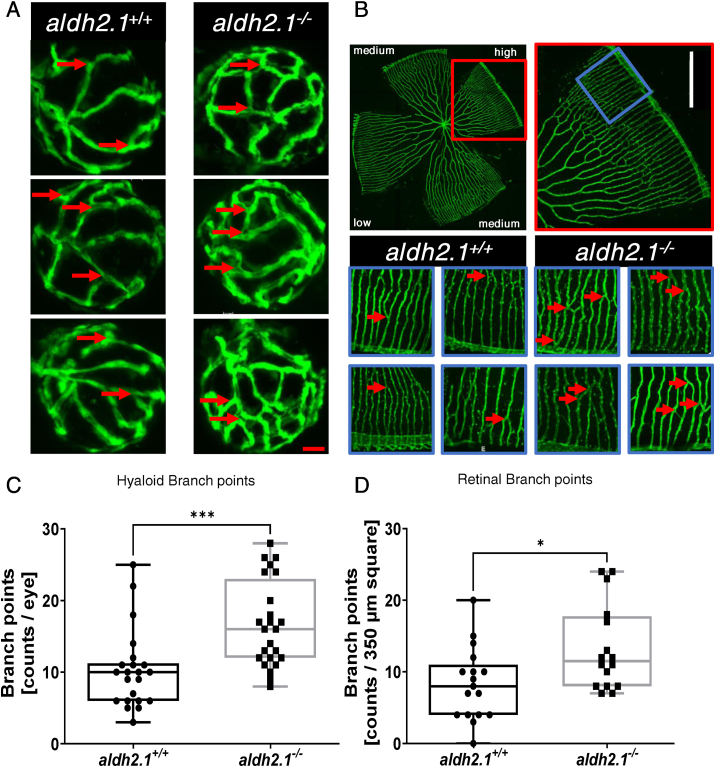
**Loss of *aldh2.1* led to increased angiogenesis in the retinal vasculature of zebrafish larvae and adults. A**: Representative confocal images of hyaloid vasculature in zebrafish larvae at 120 hpf displayed increased branching. Red scale bar: 20 μm, red arrows: branch points as counted for quantification. **B**: Representative confocal images of adult zebrafish retinae showed increased angiogenesis. White Scale Bar: 350 μm, red rectangle: high-density subdivision, blue rectangle 350 μm square, red arrows: branch points as counted for quantification. **C**: Quantification of larval hyaloid vasculature showed increased numbers of branch points in *aldh2.1*^*−/−*^ mutants, n = 22–24 eyes per group. **D**: Quantification of retinal vasculature showed increased numbers of branch points in *aldh2.1*^*−/−*^ adults, n = 16–17 350 μm squares per group, statistical analysis was done via Student's - test, *p < 0.05, ***p < 0.001. (For interpretation of the references to colour in this figure legend, the reader is referred to the Web version of this article.)

**Fig. 3:**
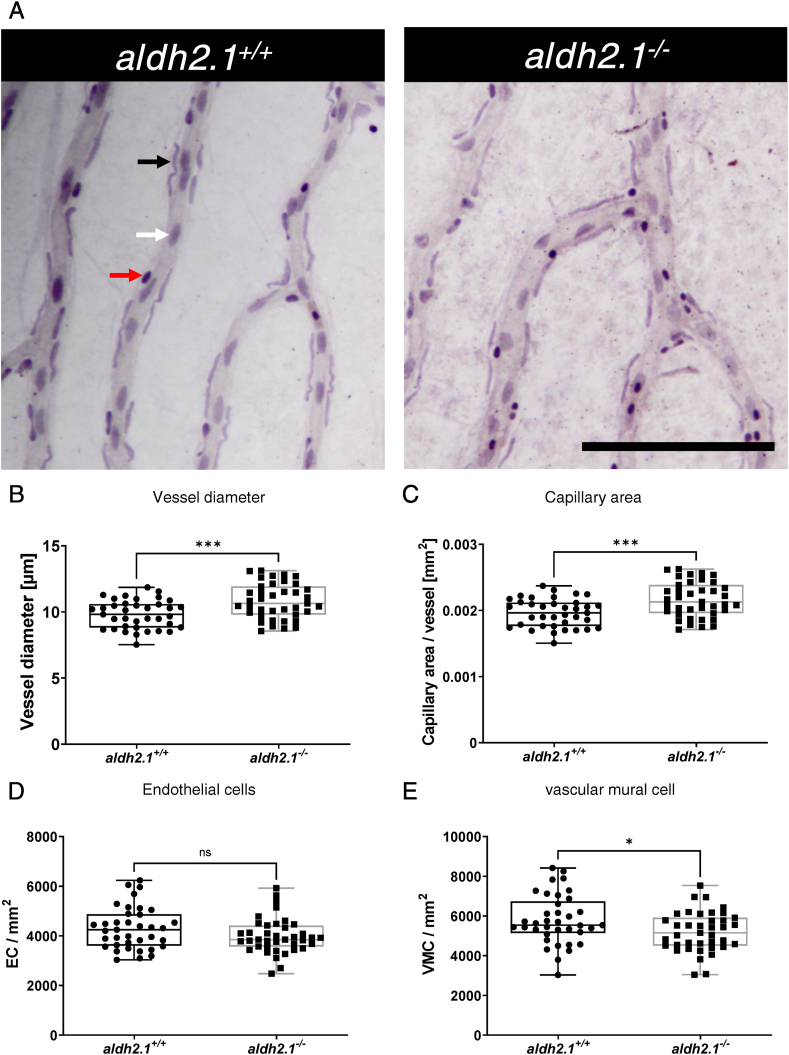
**Thickening of blood vessels and reduction of vascular mural cell coverage in *aldh2.1***^***−/−***^**retinae. A:** Representative light microscopy images of zebrafish retinae prepared with trypsin digestion and hematoxylin staining. Red Arrow: erythrocyte, black arrow: pericyte, white arrow: endothelial cell, black scale bar: 100 μm. **B-E:** Quantification of vascular parameters. **B:** Increased vessel diameter, **C:** Increased capillary area, **D:** Unaltered endothelial cell count and **E:** Reduced vascular mural cell count. n = 37–39. Statistical analysis was done via Student's t-test, ns = not significant, *p < 0.05, ***p < 0.001. (For interpretation of the references to colour in this figure legend, the reader is referred to the Web version of this article.)

In previous studies it could be shown that elevated exogenous and endogenous reactive metabolites damaged the microvasculature via an impaired glucose metabolism in zebrafish eyes [8,10,11]. To investigate a potential consequence of the *aldh2.1* knockout on vascular development, we analyzed the vasculature in larval and adult zebrafish by confocal microscopy. An increase in branch points in the hyaloid vasculature of *aldh2.1*^*−/−*^ zebrafish larvae compared to *aldh2.1*^*+/+*^ *larvae* at 5 dpf (Fig. 2A,C) could be identified. Quantification of vasculature in adult retinae at 12 mpf confirmed these results. a*ldh2.1*^*−/−*^ adults also had an increase of branch points in the high-density areas of the retina vasculature compared to *aldh2.1*^*+/+*^ adults (Fig. 2B,D). Moreover, a digest preparation of adult retinae using trypsin and a subsequent Mayer's hematoxylin staining (Fig. 3) revealed a loss of 10% vascular mural cell coverage (Fig. 3E) [26,27], with no change of endothelial cell numbers (Fig. 3D) in *aldh2.1*^*−/−*^ zebrafish vessels. Also, vessels of adult *aldh2.1*^*−/−*^ zebrafish were wider than *aldh2.1*^*+/+*^ zebrafish vessels (Fig. 3B) and subsequently had a 10% increase in capillary area per vessel (Fig. 3C).

In addition to eyes, zebrafish kidneys were also analyzed. In contrast, no apparent alterations have been found with PAS staining (Fig. S3) nor with electron microscopy (Fig. S4). These results revealed that the functional loss of Aldh2.1 led to an alteration of vasculature with similarities to patients suffering from retinopathy. Since few reports have reported an association between reactive metabolites and retinal damage [3,4,28], measurements of several reactive metabolites in larvae and adult zebrafish were performed.

### 2.3 aldh2.1^−/−^ mutants displayed an elevation of endogenous AA and decreased postprandial blood glucose

**Fig. 4:**
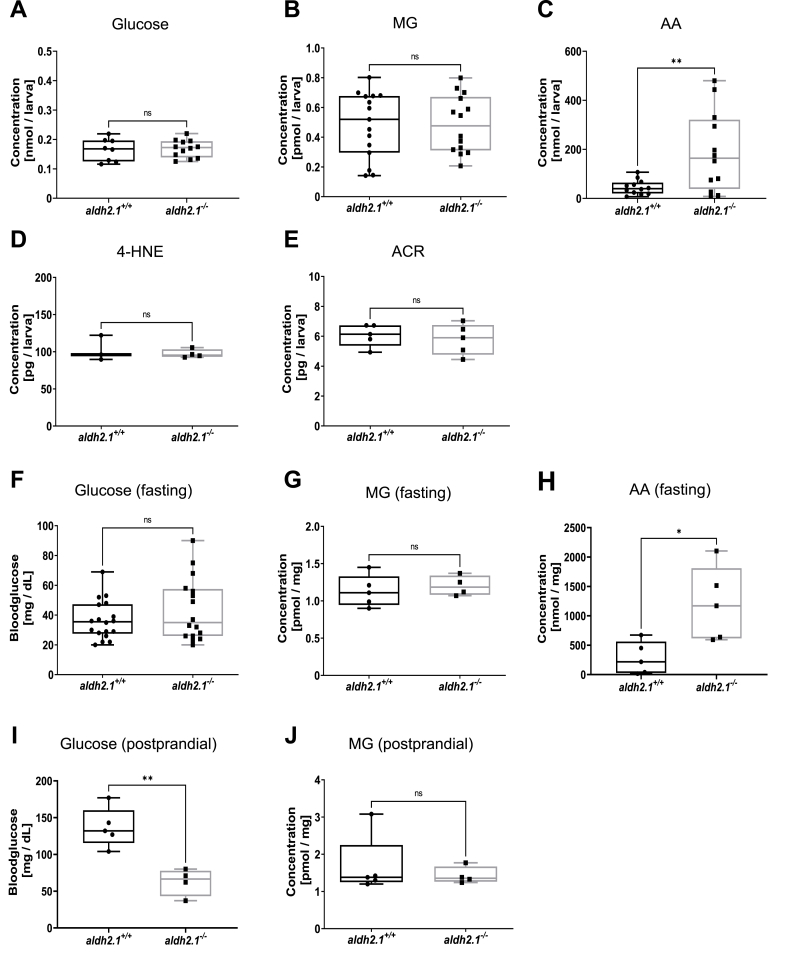
***aldh2.1***^***−/−***^**mutants displayed an elevation of endogenous AA and lowered postprandial blood glucose**. A–E: Determination of glucose and endogenous reactive metabolites in 96 hpf old zebrafish larvae displayed significantly increased AA **(C)** in *aldh2.1*^*−/−*^ mutants, but no changes for glucose (**A**), MG (**B**), 4-HNE (**D**) or ACR (**E**), n = 3–15 clutches, 50 larvae per clutch. Measurements were done via ELISA (**A,D,E**) or GC-MS/LC-MSMS (**B,C**). **F-J**: Glucose and endogenous reactive metabolites in adult zebrafish displayed significantly increased AA (**H**) in *aldh2.1*^*−/−*^ liver and decreased postprandial blood glucose (**I**). MG (**G,J**) in fasted and postprandial zebrafish eyes as well as fasting blood glucose (**F**) stayed unaltered in *aldh2.1*^*−/−*^ zebrafish, n = 4–18. Measurement of blood glucose was perfomed with a glucometer. MG and AA were measured via GC-MS and LC-MSMS respectively. Statistical analysis was done via Student's t-test, ns = not significant, *p < 0.05, **p < 0.01.

Previous studies on diabetic organ complications with the Aldh3a1 enzyme revealed an impaired RCS detoxification as the cause for the increases in angiogenesis in retinal blood vessels [10]. However, whether the impaired detoxification activity (Fig. 2G–K) in *aldh2.1*^*−/−*^ mutants translated into a similar elevation of endogenous reactive metabolites or impairment of glucose metabolism remained unknown. Thus, we conducted a series of measurements of glucose and reactive metabolites in 96 hpf old larvae and in adult zebrafish organs. In larvae, no changes in whole-body glucose (Fig. 4A), MG (Fig. 4B), Glyoxal (Fig. S6H), 4-HNE (Fig. 4D) and ACR (Fig. 4E) were found. However, we could identify an elevation of the reactive metabolite AA (Fig. 4C), which was 4.2-fold higher in *aldh2.1*^*−/−*^ larvae compared to *aldh2.1*^*+/+*^, but the metabolome remained unaltered (Fig. S5). In adult zebrafish, AA concentration was increased 4.3-fold in liver of fasted *aldh2.1*^*−/−*^ zebrafish (Fig. 4H). Yet, fasting and postprandial MG (Fig. 4G,J) were unchanged. Interestingly, while fasting blood glucose levels were unchanged in *aldh2.1*^*−/−*^ adults (Fig. 4F), postprandial measurements revealed reduced levels of blood glucose (Fig. 4I). In summary, the data have identified AA as the primary RCS of Aldh2.1's detoxification activity in zebrafish. Even though *aldh2.1* knockout has an impact on total Aldh enzyme activity for a wide spectrum of RCS (Fig. 1G–K), only AA accumulated in vivo.

### 2.4 aldh2.1^−/−^ mutants exhibited aggravated stress-signaling and an impairment of glycolysis

**Fig. 5:**
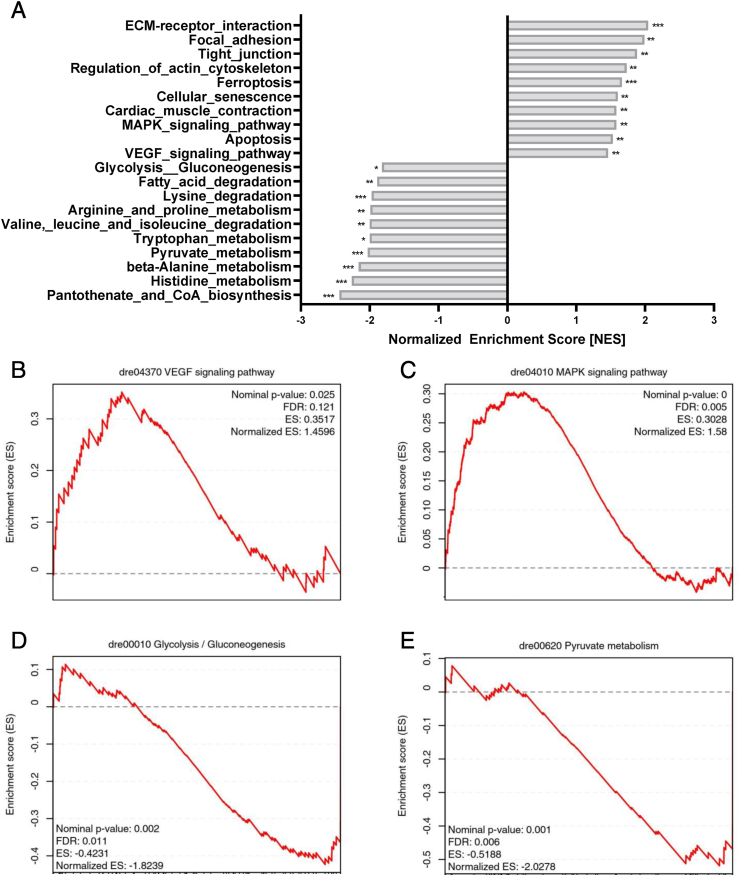
***aldh2.1***^***−/−***^**mutants exhibited aggravated stress-signaling and an impairment of glycolysis and gluconeogenesis. A**: Bar diagram for highest, significantly increased or decreased pathways in a Kyoto-encyclopedia of gene and genomes (KEGG) pathways analysis in *aldh2.1*^*−/−*^ larvae. **B-E**: Gene set enrichment analysis (GSEA) plots for VEGF (**B**), MAPK (**C**), glycolysis/gluconeogenesis (**D**), pyruvate metabolism (**E**) pathways. RNA-seq was done with mRNA in 120 hpf zebrafish larvae, quantification via normalized enrichment score (NES), n = 5 clutches, 50 larvae per clutch, ns = not significant, *p < 0.05, **p < 0.01, ***p < 0.001.

In order to investigate the underlying mechanism causing the vascular alterations in *aldh2.1*^*−/−*^ mutants and to address why *aldh2.1*^*−/−*^ mutants developed a decrease in postprandial blood glucose, we performed an RNA-sequencing analysis in zebrafish larvae with an emphasis on pathways analysis. The Kyoto-encyclopedia of gene and genomes (KEGG) pathway analysis revealed several pathways significantly regulated by the *aldh2.1* knockout, including but not limited upregulated stress signaling and a downregulated energy metabolism (Fig. 5A). Apoptosis, ferroptosis and cell senescence were among those pathways with a normalized enrichment score (NES) between 1.5 and 1.7. More importantly, overexpression of VEGF pathway components with a NES of 1.46 (Fig. 5B) and Mitogen-Activated-Protein-Kinases (MAPK) with a NES of 1.58 (Fig. 5C) were identified, providing a first hint on the mechanism in *aldh2.1*^*−/−*^ retinal blood vessels (Fig. 2). On the other site, the downregulated energy metabolism pathways included fatty acid degradation and amino acid metabolism, but also pyruvate metabolism and glycolysis/gluconeogenesis were identified (Fig. 5A). Gene set enrichment analysis (GSEA) for glycolysis/gluconeogenesis revealed a NES of −1.82 (Fig. 5D) and −2.02 for pyruvate metabolism (Fig. 5E) respectively, suggesting that *aldh2.1* is indeed involved in glucose metabolism.

### 2.5 Inhibition of glucose-6-phosphatase expression and altered expression of JNK and p38 MAPK in aldh2.1^−/−^ mutants

**Fig. 6:**
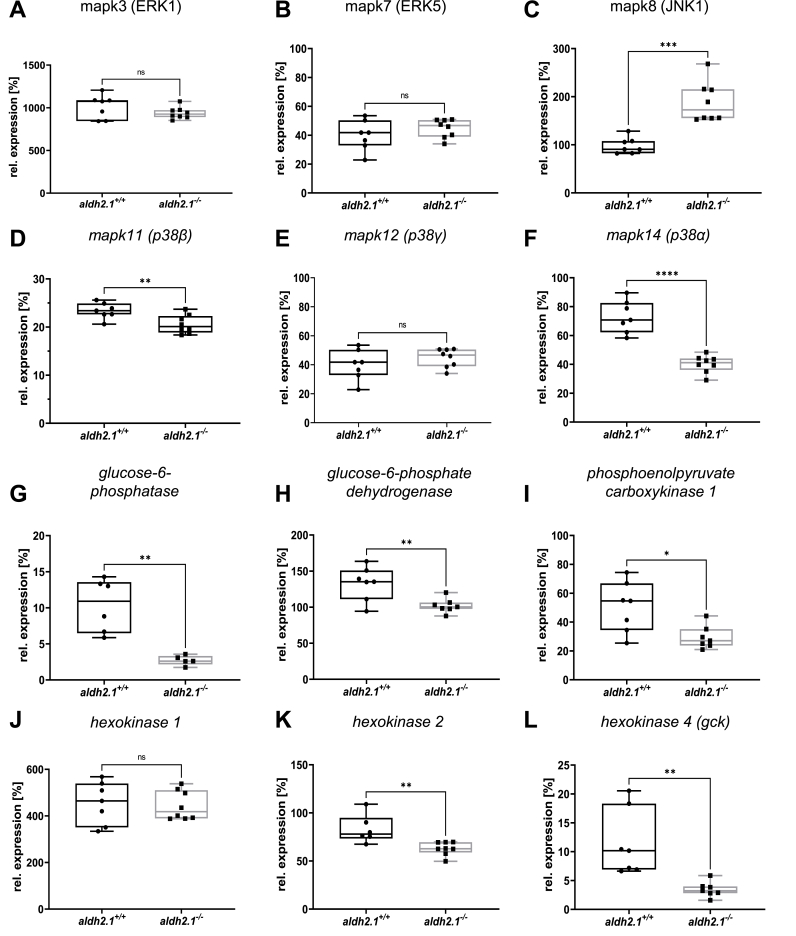
**Inhibition of glucose-6-phosphatase/glucokinase expression and alteration of JNK and p38 MAPK expression in *aldh2.1***^***−/−***^**mutants. A-F**: Expression of MAPK family members. ERK1 (**A**), ERK5 (**B**) and p38γ (**E**) were unaltered in *aldh2.1*^*−/−*^ larvae. JNK1 (**C**) is significantly upregulated; p38α (**F**) and p38β (**D**) were significantly downregulated in *aldh2.1*^*−/−*^ animals. **G-L**: Expression of key regulatory genes of glycolysis and gluconeogenesis. Strongest down regulation in *aldh2.1*^*−/−*^ larvae is shown for *g6pc* (**G**) and *gck* (**L**). *g6pd* (**H**), *pck1* (I) and *hk2 (***K**) were also significantly down regulated. *hk1* (**J**) gene expression was unchanged. Expression was quantified via RT-qPCR with 96 hpf zebrafish larvae and normalized to *arnt2*, n = 5–8 clutches, 50 larvae per clutch. Statistical analysis was done via Student's t-test, ns = not significant, *p < 0.05, **p < 0.01, ***p < 0.001, ****p < 0.0001.

Based on the RNA-seq data (Fig. 5), we explored expression of selected genes within the identified pathways which may explain the underlying mechanism of *aldh2.1*^*−/−*^ zebrafish related to increased retinal angiogenesis and impaired glucose homeostasis. Gene expression of angiogenesis marker, such as *fgfr2, vegfr2* and *notch1a* [29] were unchanged between *aldh2.1*^*−/−*^ and *aldh2.1*^*+/+*^ larvae (Figs. S6E–G). Therefore, the focus was then changed to members of the MAPK family, since ERK1, JNK and p38 MAPK are all known for their abilities to alter endothelial cell activation and angiogenesis [[30], [31], [32], [33]]. *mapk3* and *mapk7* also known as ERK1 (Fig. 6A) and ERK5 (Fig. 6B) respectively, were not changed. However, *mapk8b,* also known as JNK1, revealed a two-fold increase in expression (Fig. 6C) while *mapk11-14*, known as p38 MAPKs exhibited an up to two-fold decrease in expression (Fig. 6D,E,F). Simultaneously, we explored the cause for lowered postprandial blood glucose concentrations in adult *aldh2.1*^*−/−*^ mutants with selected genes regulating important steps in glycolysis, gluconeogenesis and glucose internalization. RT-qPCR identified a four-fold decrease of glucose-6-phosphatase (*g6pc*, Fig. 6G) expression, which in gluconeogenesis converts glucose-6-phosphate into glucose. Intriguingly, a single report has indeed hypothesized several years ago that AA suppresses glucose-6-phosphatase expression [34] and our data now show this regulation for the first time in vivo since AA is highly increased in the *aldh2.1*^*−/−*^ mutant (Fig. 4C,H). Correspondingly, a three-fold decrease of glucokinase (*gck)* (Figure 6L), that mediates the first step in glycolysis by phosphorylation of glucose to glucose-6-phosphate, was observed in *aldh2.1*^*−/−*^ mutants. Moreover, phosphoenolpyruvate decarboxylase expression (*pepck)* (Fig. 6I), a key regulator of gluconeogenesis, was downregulated 1.7-fold and glucose-6-phosphate dehydrogenase (*g6pd)* (Fig. 6H), which is stimulated by its substrate – glucose-6-phosphate, was also downregulated 1.3-fold. Finally, insulin expression as well as *pdx 1* expression in *aldh2.1*^*−/−*^ mutant larvae was significantly downregulated (Figs. S6C and D). Together, the selected single gene expression data confirmed the RNA-seq data and they may explain the increased angiogenesis in retinal vessels of *aldh2.1*^*−/−*^ mutants via altered JNK and p38 MAPK signaling in zebrafish larvae. Furthermore, this data also suggests a new mechanism of impaired glucose metabolism in which an increase of endogenous AA, which inhibits glucokinase and glucose-6-phosphatase expression, causes hypoglycemia.

### 2.6 Exogenous AA caused angiogenic alterations in hyaloid vasculature, impairment of glucose metabolism and alteration of MAPK signaling

**Fig. 7:**
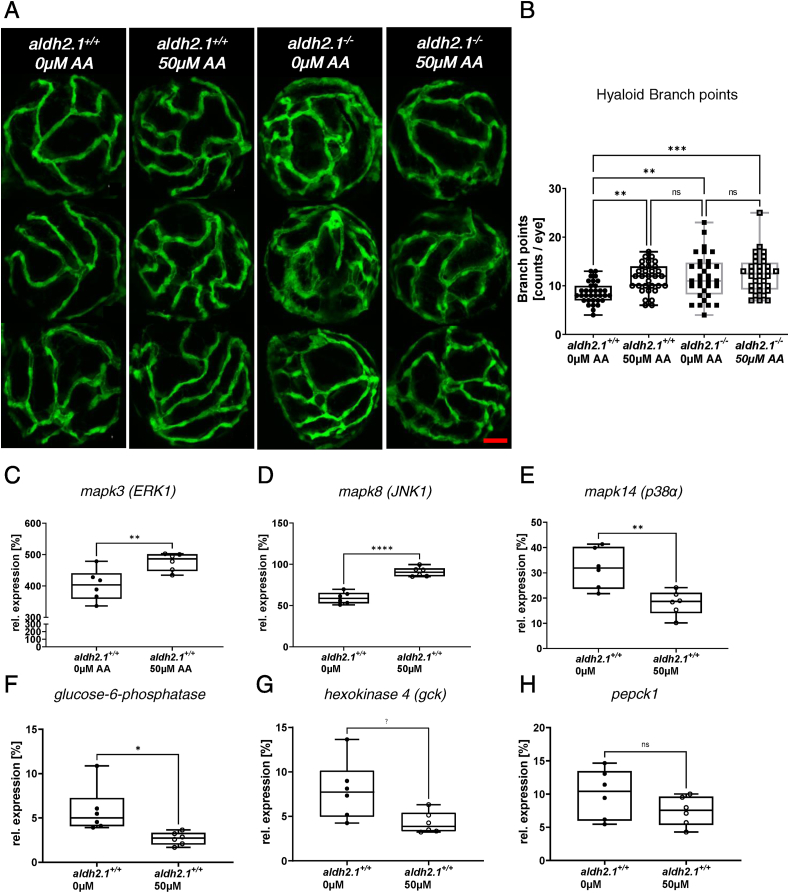
**Exogenous AA caused angiogenic alterations in hyaloid vasculature, impairment of glucose metabolism and alteration of MAPK signaling. A**: Representative confocal images of hyaloid vasculature in zebrafish larvae at 120 hpf with and without AA treatment. Red scale bar: 20 μm. **B**: Quantification of larval hyaloid vasculature showed increased numbers of branch points in *aldh2.1*^*+/+*^ larvae after AA (50 μm) treatment, which was not further enhanced in *aldh2.1*^*−/−*^ larvae, n = 32–33 eyes per group. **C-E**: Expression of MAPK family members. ERK1 gene expression (**C**) was increased in *aldh2.1*^*+/+*^ larvae after AA treatment. Additionally, *aldh2.1*^*+/+*^ larvae with 50 μm AA treatment exhibited increased JNK1 expression (**D**) and decreased p38α expression (**E**). **F–H**: Expression of selected genes of glycolysis and gluconeogenesis. Reduced expression of *g6pc* (**F**) and *gck* (**G**) after AA treatment. *pepck* gene expression (**H**) was also reduced, although not significantly. Expression was quantified via RT-qPCR with 96 hpf zebrafish larvae and normalized to *arnt2*, n = 6 clutches, 50 larvae per clutch. Statistical analysis was done via Student's t-test, ns = not significant, *p < 0.05, **p < 0.01, ***p < 0.001, ****p < 0.0001. (For interpretation of the references to colour in this figure legend, the reader is referred to the Web version of this article.)

Analysis of *aldh2.1*^*−/−*^ mutants identified microvascular complications in retinal vessels, but it remained unclear whether these alterations were directly induced by the *aldh2.1*^−/−^ mutant or indirectly by increased endogenous AA and subsequently impairing glucose metabolism and stress signaling. In order to address this question, we incubated wildtype zebrafish larvae with AA and repeated prior analysis of retinal hyaloid structures and RT-qPCR for selected gene expressions. Toxicity tests for AA in zebrafish larvae were done beforehand and revealed a tolerance of up to 500 μM AA (Fig. S1). Analysis of hyaloid vessels in 120 hpf old larvae incubated with 50 μM AA showed that exogenous AA can mimic the microvascular complications as seen in *aldh2.1*^*−/−*^ mutants, but does not amplify it. Between *aldh2.1*^*+/+*^ zebrafish larvae with and without AA incubation there is a 1.36-fold increase of branch points. Branch points between *aldh2.1*^*+/+*^ without exogenous acetaldehyde and *aldh2.1*^*−/−*^ with and without AA treatment were also increased by 1.3–1.5-fold factor (Fig. 7A,B). Additional to retinal analysis, RT-qPCR was performed with AA incubated wildtype zebrafish larvae. Results displayed a 1.53-fold upregulation of JNK1 (Fig. 7D) expression and a 1.77-fold downregulation of p38α MAPK (Fig. 7E) expression. Additionally, ERK1 (Fig. 7C) also shows a slight increase in expression. Intriguingly, after AA treatment g*6pc* (Fig. 7F) and *gck* (Fig. 7G) expression decreased by factor 2.18 and 1.84, while *pepck* (Fig. 7H) was not significantly reduced. In conclusion, the data show that impaired glucose metabolism in *aldh2.1*^*−/−*^ mutants is caused by AA. Due to the loss of *aldh2.1,* AA is not detoxified and accumulates and downregulates *gck* and *g6pc*. In addition, AA was also identified as the driver of increased angiogenesis in *aldh2.1*^*−/−*^ retinal vasculature.

### 2.7 Inhibition of p38 MAPK caused similar, but not identical angiogenic alterations in hyaloid vasculature in aldh2.1^+/+^ zebrafish larvae

**Fig. 8:**
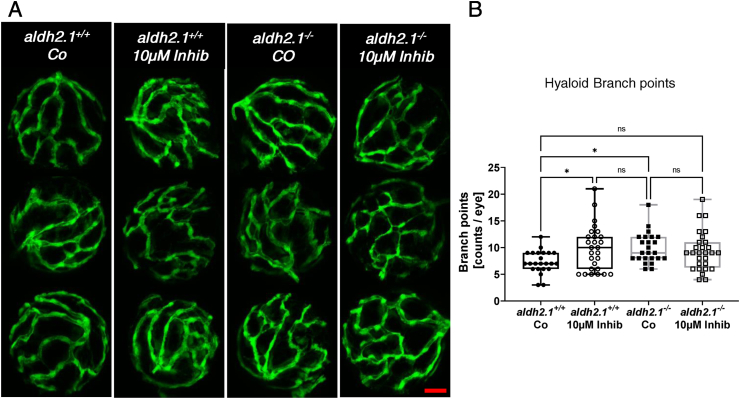
**Inhibition of p38 MAPK caused angiogenic alterations in hyaloid vasculature in *aldh2.1***^***+/+***^**larvae. A**) Representative confocal images of hyaloid vasculature in zebrafish larvae at 120 hpf with and without MAPK inhibitor treatment. Red scale bar: 20 μm. **B**) Quantification of larval hyaloid vasculature showed increased amount of branch points in *aldh2.1*^*+/+*^ larvae with MAPK inhibitor comparable to *aldh2.1*^*−/−*^ mutants, n = 23–28 eyes per group. Statistical analysis was done via Student's t-test, ns = not significant, *p < 0.05. (For interpretation of the references to colour in this figure legend, the reader is referred to the Web version of this article.)

To finally prove that p38 MAPK is the missing link between increased AA levels, *aldh2.1* knockout and microvascular complications, zebrafish larvae were incubated with the selective p38 MAPK inhibitor 4-(4-Fluorophenyl)-2-(4-methylsulfinylphenyl)-5-(4-pyridyl)1H-imidazole. Analysis of hyaloid vessels (Fig. 8A,B) was performed with p38 MAPK inhibitor incubated *aldh2.1*^*+/+*^ and *aldh2.1*^*−/−*^ zebrafish larvae and displayed similar results as prior studies on *aldh2.1*^*−/−*^ mutants (Fig. 2) and larvae treated with AA (Fig. 7). *aldh2.1*^*+/+*^ zebrafish hyaloid vasculature exhibited a 1.34-fold increase in branch points after treatment with p38 MAPK inhibitor. Treatment of *aldh2.1*^*−/−*^ mutants could not increase branch point formation any further. This is in line with previous RT-qPCR results, as p38 MAPK mRNA expression is already significantly downregulated in *aldh2.1*^*−/−*^ mutants. In summary, the experiments suggest, that *aldh2.1*^*−/−*^ knockout leads to an increased retinal angiogenesis in the eyes of zebrafish via imbalance of MAPKs including but not limited to loss of p38 MAPK function induced by AA. Lastly, because incubation with p38 MAPK inhibitor did only result in a non-significant increase of hyaloid vessel branch points in *aldh2.1*^*−/−*^ knockout larvae compared to *aldh2.1*^*+/+*^ larvae, it is suggested that p38 MAPK is not the sole driver of this phenotype.

## 3 Discussion

In this study, we established an *aldh2.1*^*−/−*^ mutant zebrafish model to study RCS detoxification and the consequences of accumulated endogenous AA in vivo. Increased AA led to microvascular damage in retinal blood vessels and caused lowered postprandial blood glucose levels by blocking *gck* and *g6pc* gene expression. Thus, the study identified a novel mechanism how RCS impair the glucose metabolism and induce angiogenesis without involving hyperglycemia.

RCS are spontaneously formed in the metabolism and are considered as dangerous molecules because they can modify and impair the function of DNA, proteins and lipids [24,[35], [36], [37], [38], [39]] In recent years, it was shown that the loss of a specific RCS detoxifying enzyme led to an increase of a preferred RCS, which subsequently altered the glucose metabolism and mediated the development of diabetic organ complications. Specifically, loss of *glo1* in zebrafish increased MG concentrations and was accompanied by an impaired glucose tolerance [8]. In *aldh3a1* knockout zebrafish mutants, increased 4-HNE concentrations disrupted the pancreas formation, which inhibited insulin expression and thereby facilitated hyperglycemia and a retinal vasodilatory phenotype [10]. In addition, increased ACR in *akr1a1a* zebrafish mutants led to insulin resistance and consequently to diabetic retinopathy and diabetic nephropathy [11]. In the present study, we now show that increased AA concentration in *aldh2*.*1*^*−/−*^ mutant zebrafish impaired glucose metabolism causing decreased blood glucose levels and induced an activation of angiogenesis. Together, the data have identified a specific signature of individual RCS and their corresponding detoxifying enzyme systems, causing a specific organ pathology ranging from altered glucose metabolism, impaired glucose tolerance, loss of pancreatic insulin expression, insulin resistance to hyperglycemia. All accumulated RCS, except for AA, caused hyperglycemia, which led to the hallmarks of diabetic retinopathy or diabetic nephropathy. AA also induces microvascular alterations in the retina, but independent of hyperglycemia.

Further experiments then identified that AA causes an impaired glucose sensitivity and glucose mobilization via blocking expression of key regulatory enzymes of glycolysis and gluconeogenesis, namely *gck* and *g6pc,* which ultimately led to lowered postprandial blood glucose concentrations in *aldh2.1*^*−/−*^ mutants. The human homolog of *aldh2.1, aldh2*, has already been extensively studied for its importance of ethanol detoxification. However, research on alcohol mediated stress complications mainly comprise of cardiac disease, cancer and liver disease, whereas studies on *aldh2* and metabolomic diseases, such as diabetes mellitus, are scarce [17,18,21,40,41]. Intriguingly, observations made in diabetic patients already connected alcohol consumption and hypoglycemic episodes, but the underlying mechanism on how alcohol inhibits both glycolysis and gluconeogenesis remained unexplored [[42], [43], [44]]. This study now provided an explanation that the important upstream factor causative for this phenomenon is not ethanol directly, but AA. AA blocks *g6pc* expression, a finding that has already been hypothesized several years ago [34] and subsequently inhibits formation of glucose from glucose-6-phosphate and its release into the blood stream. Moreover, AA also induced a decrease in glucose sensitivity by blocking expression of *gck.* The latter itself is already known for - if mutated - causing diabetes or hypoglycemia [[45], [46], [47]]. Thereby, this study suggests a high relevance for *aldh2* and AA in regulating glucose metabolism [[48], [49], [50], [51]].

This study also showed that increased endogenous AA in *aldh2.1*^*−/−*^ mutants led to retinopathy without hyperglycemia. Aldh2 is known to have an important function in the protection of mitochondria, specifically loss of *aldh2* triggers increase of ROS formation through CYP2E1, Nrf2 and TNF-α [17,21,52,53] and subsequently RCS [54,55]. Additionally, altered Aldh2 and correspondingly AA correlate with cardiovascular diseases including but not limited to ischemia and myocardial dysfunction [[56], [57], [58], [59]] Nevertheless, the mechanism for the developed retinopathy in *aldh2.1*^*−/−*^ zebrafish without hyperglycemia had yet to be described. Unbiased expression data in combination with a pharmacological intervention study illuminated the mechanism and showed an imbalance of JNK and p38 MAPK expression in *aldh2.1*^*−/−*^ zebrafish. Both stress-activated protein kinases are well known to have multiple functions in angiogenesis; on one side they act as molecular switches for angiogenesis through endothelial cell activation; additionally they can induce hyperpermeability in endothelial cell layers. [32,33,[60], [61], [62], [63]]. Furthermore, vascular mural cell dropout has long been theorized to be a key player in microvascular homeostasis and recent studies have shown that loss of mural cells increases the susceptibility of the retinal vasculature to VEGF signaling and retinal angiogenesis [26,[64], [65], [66]]. Thus, the onset of retinopathy in *aldh2.1*^*−/−*^ larvae and adults was driven by an altered regulation of the MAPK family members and reduction of vascular mural cell coverage through the reactive metabolite AA independently of hyperglycemia.

In summary, the data have several important implications and raise a couple of questions in diabetes research and clinical translation about RCS. First, this study and previous data show that internally produced RCS in the metabolism can cause an impaired glucose metabolism, diabetes and diabetes related organ complications [8,10,11]. Second, the data suggest that as long as the RCS corresponding enzyme systems are functional, diabetes and microvascular organ alterations can be prevented. Third, it is urgent to identify upstream factors of the detoxifying enzyme system and understand their mechanisms of activation and how they are altered in different disease conditions. Fourth, the interplay and crosstalk of RCS and their corresponding enzyme systems in diabetes, but also in other diseases, must be identified. And last, it seems now more than essential to address the question whether the identified signature of altered RCS in diseased zebrafish also exists in human diseases. It must be investigated whether RCS can be used as biomarkers for the different subtypes of diabetes [67] and whether they induce the same diabetic complications in humans as they were identified in zebrafish.

## 4 Material and methods

### 4.1 Study approval

All experimental procedures on animals were approved by the local government authority Regierungspräsidium Karlsruhe and by Medical Faculty Mannheim (G-98/15 and I-21/04) and carried out in accordance with the approved guidelines.

### 4.2 Zebrafish husbandry

In this study the zebrafish line *Tg(fli1:EGFP)* was used [68]. Embryos/larvae were held and raised in egg water at 28.5 °C for 144 h before being transferred to adult boxes. Adult Zebrafish were kept under a 13-h light/11-h dark cycle. Fish older than 72 h-post-fertilization (hpf) are referred to as larvae, after 90 days-post-fertilization (dpf) they are referred to as adults [69]. Feeding of Zebrafish took places twice a day, freshly hatched Artemia Salina in the morning and fish flake food in the afternoon.

### 4.3 Mutant generation

The *aldh2.1*^−/−^ fishline was generated using CRISPR/Cas9 as previously described. Briefly: the technique used one guide RNA (gRNA) targeting exon 3 of *aldh2.1*, which was designed using ZiFiT Targeter 4.2 and cloned into a T7-driven promoter expression vector (pT7-gRNA; Addgene) (Table S2). Additionally the pT3TS-nCas9n Vector (Addgene) was used in vitro for transcription to attain Cas9 mRNA [70]. Following the protocol of the manufacturer for mRNA Synthesis, the mMESSAGE mMACHINE T3 Transcription Kit and the MEGAshortscript T7 Kit (Invitrogen) were used for Cas9 mRNA and gRNA respectively. Afterwards a solution of KCl (0.1 m) containing gRNA (200 pg/L) and Cas9 mRNA was injected into one-cell stage Zebrafish embryos [70]. The resulting adult mosaic zebrafish (F0) were analyzed for germline transmission via Sanger sequencing of PCR products (Table S2) and selectively bred. Mutations were identified by evaluation of the chromatograms and use of Yost tools Poly Peak Parser [71].

### 4.4 Preparation of adult zebrafish and blood glucose measurement

Adult Zebrafish were transferred to single boxes and fasted one day prior to preparation. After 16–18 h, fish were used either directly or first fed with 0.5 g flakes for 1 h followed by another hour in fresh water for postprandial measurement. Before preparation and blood glucose measurement could take place, fish were euthanized in ice water for 2 min. Subsequently blood was extracted from caudal vessels and measured by a glucometer (Freestyle Abbott) [72]. Immediately after, the fish were transferred to an experimental platform covered with ice-cold PBS. For Metabolomics, RT-qPCR or Western Blot analysis organs were isolated, transferred, weighed and snap frozen in liquid nitrogen and then stored at −80 °C. Alternatively, for Visualization via either Confocal Microscopy or Histology organs were isolated and transferred into 4% PFA/PBS for at least 24 h before further analysis. Lastly, for Electron Microscopy kidneys were isolated and transferred into 3% Glutaraldehyde in Cacodylate (0.1 m) before further handling.

### 4.5 Microscopy and analysis of vascular alterations in larvae and adults

Confocal images for phenotype evaluation were acquired using a confocal fluorescence microscope (DM6000 B) with a scanner (Leica TCS SP5 DS) utilizing a 20 × 0.7 objective, 1024× 1024 pixels and 1.5 μm Z-steps. For evaluation Leica Application Suite X, Gimp 2 and ImageJ were used.

Alterations in larvae retinal hyaloid vasculature was imaged at 5 dpf, at which point larvae were anaesthetized in 0.003% tricaine and fixed in 4% PFA/PBS for 24 h at 4 °C. Fixed larvae were washed three times for 15 min in PBS at RT before incubation in 0.25% Trypsin/EDTA solution (Gibco) buffered at pH 7.8 with TRIS (1.5 m, Roth) for 80 min at 37 °C. Afterwards larvae were washed three times for 15 min and stored in PBS until preparation. The larvae retinal hyaloid vasculature was dissected under a stereoscope and visualized with the confocal fluorescence microscope as described above [73].

For imaging of the zebrafish adult retinal vasculature, retina dissection and analysis were performed as recently described [74]. Fixated eyes were transferred to an agarose platform covered with PBS. The retina was detached from the eye and washed before it was transferred on a slide, immersed in mounting media and covered with a cover slide. Pictures were taken using the confocal fluorescence microscope as described above.

### 4.6 Retinal digest preparation

Retinal digest preparations were performed according an established protocol with slight alterations [75]. After extracting the eye from the zebrafish head it was transferred into 4% formalin for fixation for 48 h. After fixation, the retina was dissected according to the protocol mentioned above [74]. After dissection the retina was transferred into ddH_2_O and incubated at 37 °C overnight. It was then transferred into 3% porcine trypsin (Sigma-Aldrich) in Tris-HCl (0.2 m) and incubated again at 37 °C for 1.5 h. After incubation in trypsin the retina was transferred to a microscope slide and the retinal cells were removed from the vasculature by dropping ddH_2_O from a syringe on top of the retina. The cells were removed from the slide through water aspiration and the vasculature was left to air-dry.

The retinal digest preparations were stained using Mayer's hemalum solution (Millipore). The slides were briefly placed in ddH_2_O and then moved to fresh undiluted Mayer's hemalum solution for 7 min. Afterwards, they were placed in lukewarm tap-water for 2 min and then moved shortly to 70% and then 80% ethanol before being put in first 96% and then 99.8% ethanol and kept there for 5 min each. After the last change of ethanol, the slides were placed in two changes of xylene and kept there for 5 min each before the slides were covered with cover slips using DPX mounting medium (Thermo Fisher Scientific).

Images of the stained digest preparations were taken at 200× magnification using the B×51 upright microscope (Olympus Life Science) with the XC10 camera (Olympus Life Science). Analysis of the vessel diameter and the number of endothelial cells and pericytes was performed using the Cell-F software (Olympus Opticals). The cells were counted in six to eight randomly selected areas in a circular area of the intermediate third of the retina, leaving out the area close to the entrance of the optic artery into the retina and the peripheral area. Endothelial cells and pericytes were identified by their distinct location and morphology. The cells were counted over 200 μm and the vessel diameter was measured. The cell numbers were then calculated as number of cells per mm^2^ of capillary area.

### 4.7 Analysis of kidney morphology

Imaging and Processing of zebrafish kidneys for Electron Microscopy (EM) was prepared in cooperation with the institute of Pathology IPH at Heidelberg University Hospital. Preparation was done as previously described [11].

### 4.8 Western blot analysis

For Western blot analysis, larvae/adult organs were taken, incubated for 10 min with Natrium-Vanadate (2 mm) in 1 × PBS on ice to inhibit phosphatases. Then they were lysed in NP40 lysis buffer (NaCl (150 mmol L^−1^), Tris-HCl (50 mmol L^−1^), pH 7.4, 1% NP40, EDTA (10 mmol L^−1^), 10% glycerol, and protease inhibitors) using a 1 mL Syringe and a 25 G needle. Followed by incubation on ice for 30 min on a shaker. The supernatant containing the protein lysate was diluted 5:1 with Laemmli sample buffer and boiled at 95 °C for 5 min, separated via SDS-PAGE, and then transferred to a nitrocellulose membrane for antibody incubation. Visualization by enhanced chemiluminescence (ECL) was acquired after incubation with Horseradish Peroxides substrate (HRP, Supersignal™ Thermo Scientific).

### 4.9 Antibody generation

For zebrafish Aldh2.1 antibody generation, peptide (QHSTIPAPNVQPDVHYNKIC) was designed, synthesized, and coupled to KLH (Keyhole Limpet Hemocyanin) by PSL GmbH, Heidelberg, Germany and subsequently injected into guinea pigs for immunization following standard procedures from GPCF Unit Antibodies, DKFZ Heidelberg, Germany.

### 4.10 Pharmacological treatment of zebrafish embryos/larvae

Fertilized zebrafish embryos were transferred into 5 cm petri dishes. Each Petri dish held 30 embryos in 10 mL egg water. At 24 hpf, the chorion was removed using tweezers. Treatment with either AA (10 μm–5 mm, Sigma-Aldrich) or p38 MAPK inhibitor (10 μm,Calbiochem, SB 203580) started at 4 hpf and was refreshed daily at 24, 48, 72, 96 hpf.

### 4.11 Collection of zebrafish embryo/larvae sample

If not separately, specified embryo and larvae samples were anaesthetized with 0.003% tricaine in different developmental time points between 24 hpf and 120 hpf, collected and snap frozen in liquid nitrogen in a clutch of 50. Prior to each assay, zebrafish larvae were homogenized in assay buffer using a 1 mL syringe with a 25 G needle.

### 4.12 Enzyme activity assay

Total ALDH activity was measured with larvae at 96 hpf at 25 °C in Tris-HCl (0.5 mm, pH 9.5) containing DL-2-amino-1-propanol (10 mm), NAD (0.5 mm) and one of the following: MG (2 mm) or 4-HNE (4 mm) or AA (5 mm) or MDA or ACR by measuring the rate of NAD formation at 340 nm [24]. Glo 1-activity was determined spectrophotometrically monitoring the change in absorbance at 235 nm caused by the formation of S-d-lactoylglutathione [76].

AKR activity was determined by measuring the rate of reduction of NADPH at 340 nm, pH 7.0, and 25 °C. The assay mixture contained potassium phosphate (100 mm), DL-glyceraldehyde (10 mm)/ACR (5 mm), and NADPH (0.1 mm).

### 4.13 Whole body glucose

Glucose content was determined according to the manufacturer's instruction (Glucose Assay Kit, CBA086, Sigma-Aldrich) with 96 hpf larvae lysates.

### 4.14 Methylglyoxal assay

MG, 3-DG, and glyoxal were measured as previously described [8,77,78].

### 4.15 Acetaldehyde assay

AA was measured in a LC-MSMS experimental setup, using acetaldehyde-d4 as internal standard [79].

### 4.16 4-HNE assay

4-HNE amount was determined according to the manufacturer's instruction (4-Hydroxynonenal ELISA Kit, E4645, Biovision) with 96 hpf larvae lysates.

### 4.17 Acrolein assay

Protein-bound ACR was determined according to manufacturer's instruction (Acrolein ELISA Kit, MBS7213206, MyBioSource Inc) with 96 hpf larvae lysates.

### 4.18 Reverse-transcription quantitative polymerase chain reaction (RT-qPCR)

Total RNA was isolated from homogenized zebrafish larvae (96 hpf/120 hpf) or organs using RNeasy Mini Kit according to the manufacturer's instruction (Qiagen). Subsequently cDNA was generated following the instructions Maxima First Strand cDNA Synthesis Kit (Thermo Scientific). Primer design was done using the NCBI primer blast tool and are listed in Table S3. RT-qPCR was done with PowerSYBR™ Green PCR Master (Thermo Scientific) Mix in 96 - well reaction plates. The qPCR reaction was performed with Quant Studio 3 Real-Time-PCR-System.

### 4.19 RNA-seq analysis

Total RNA was isolated from homogenized zebrafish larvae (120 hpf) or organs using RNeasy Mini Kit according to the manufacturer's instruction (Qiagen). Library construction and sequencing were performed with BGISEQ-500 (Beijing Genomic Institution, www.bgi.com, BGI). Gene expression analysis were conducted by the Core-Lab for microarray analysis, centre for medical research (ZMF) as previously described [10]. The data is available on https://www.ncbi.nlm.nih.gov/geo/query/acc.cgi?acc=GSE189416.

### 4.20 Metabolomic analysis

Detection was done in cooperation with the Metabolomics Core Technology Platform from the Centre of Organismal Studies Heidelberg. Adenosine compounds, thiols, free amino acids, fatty acids and primary metabolites were measured as previously described [8].

### 4.21 Protein sequence alignment

The amino acid sequences of Aldh2.1 protein from zebrafish, human and mouse were accessed by Uniprot database. For comparison the selected sequences were aligned using Clustal Omega Multiple Sequence Alignment (https://www.ebi.ac.uk/Tools/msa/clustalo/).

### 4.22 Statistics

Experimental results are expressed median using box plots with whiskers. Statistical significance between different groups was analyzed using Student's t-test. GraphPad Prism 8.3.0 was used for analyses and p values of 0.05 were considered as significant: *p < 0.05, **p < 0.01, ***p < 0.001, ****p < 0.0001.

## Author contributions

D.P.W. performed experiments, analyzed data and wrote the manuscript. B.L. generated *aldh2.1* knockout zebrafish, C.S.M. performed retina digests and analysis. J.M. and T.F. implemented and performed biochemical experiments and gave conceptual and technological advice. C.S. analyzed RNA-seq data. I.H. performed histological analysis and electron microscopy of adult zebrafish kidneys. J.S., R.H., H.P.H. and P.P.N. gave conceptual and technological advice and revised the manuscript. J.K. conceived and designed the study and wrote the manuscript. J.K. is the guarantor of this work and has full access to all data and takes responsibility for the integrity of the data and accuracy of the data analysis.

## Declaration of competing interest

The authors declare no conflict of interest.
