# Supplementary material for: Accumulation of acetaldehyde in aldh2.1−/− zebrafish causes increased retinal angiogenesis and impaired glucose metabolism
Source: Redox Biol. 2022 Jan 26;50:102249. doi: 10.1016/j.redox.2022.102249 (PMC8818574; doi:10.1016/j.redox.2022.102249)
Supplement: Multimedia component 1 [file mmc1.pdf]

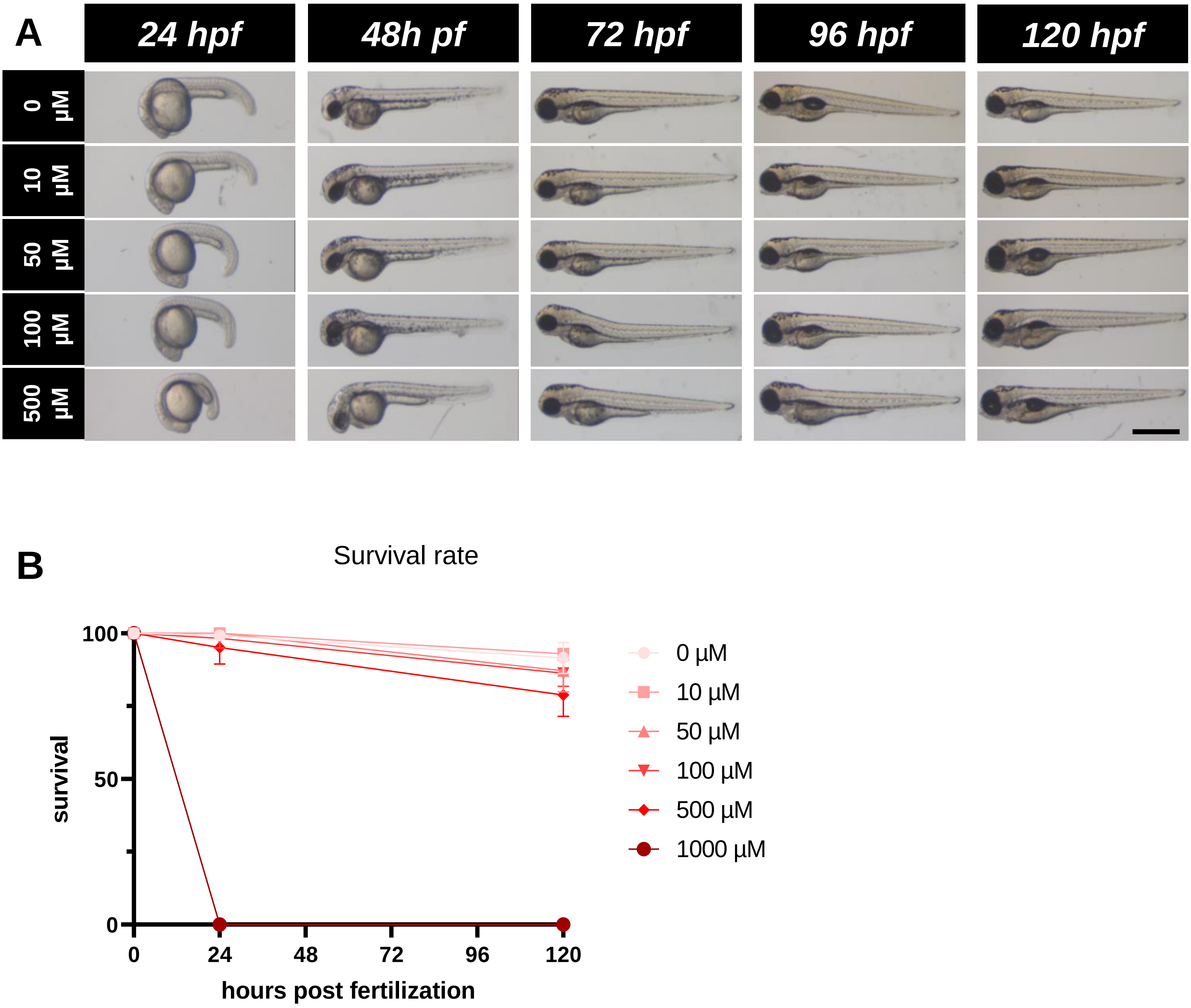

Figure S1. Zebrafish larvae displayed no morphological alterations at 120 hpf after incubation with up to 500 µM AA. A: Representative microscopic images of zebrafish larvae between 24 hpf and 120 hpf displayed normal development up to 500 µM AA treatment. Black scale bar: 500 µm. **B:** Quantification of survival rates showed high lethality of 1000 µM AA treated larvae.

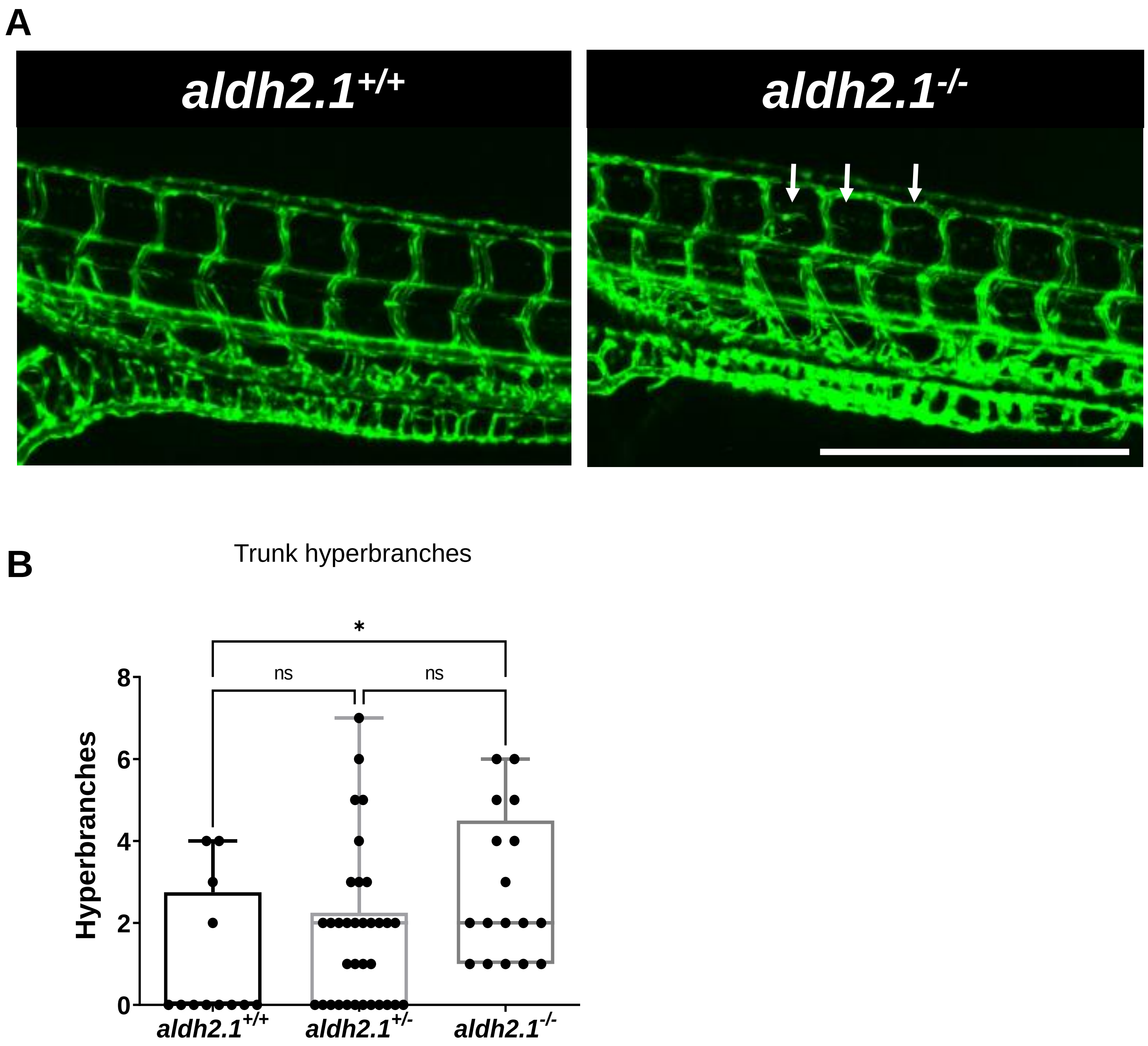

Figure S2. Trunk angiogenesis was enhanced in *aldh2.1*$^{-/-}$ larvae. (A): Representative images of the trunk vasculature in zebrafish larvae at 120 hpf displayed increased hyperbranching. White scale bar: 500 µm, white arrows: hyperbranches. **B**: Quantification of hyperbranches revealed a significant increase in hyperbranches between *aldh2.1*$^{+/+}$ and *aldh2.1*$^{-/-}$ larvae. Statistical analysis was done via Student's t-test, ns = not significant, *$p < 0.05$.

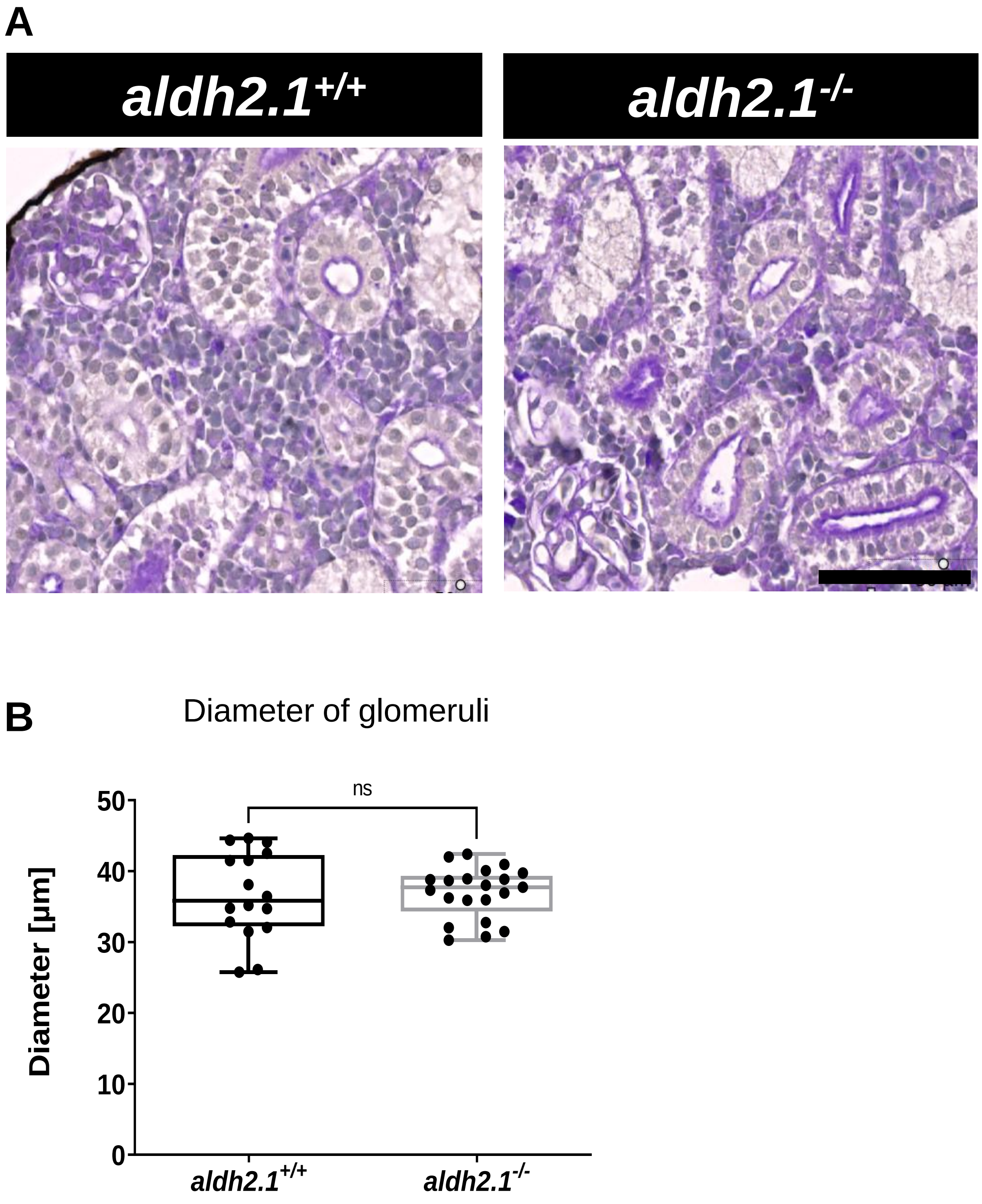

Figure S3. Unaltered renal morphology in adult *aldh2.1*$^{-/-}$ zebrafish. A: Representative Periodic acid-Schiff (PAS) staining showed no alterations in gross morphology of *aldh2.1*$^{-/-}$ kidneys. Black scale bar: 50 µm. **B:** Quantification of glomerular diameter in zebrafish kidneys showed no changes between *aldh2.1*$^{+/+}$ and *aldh2.1*$^{-/-}$ zebrafish. Statistical analysis was done via Student's t-test, ns = not significant.

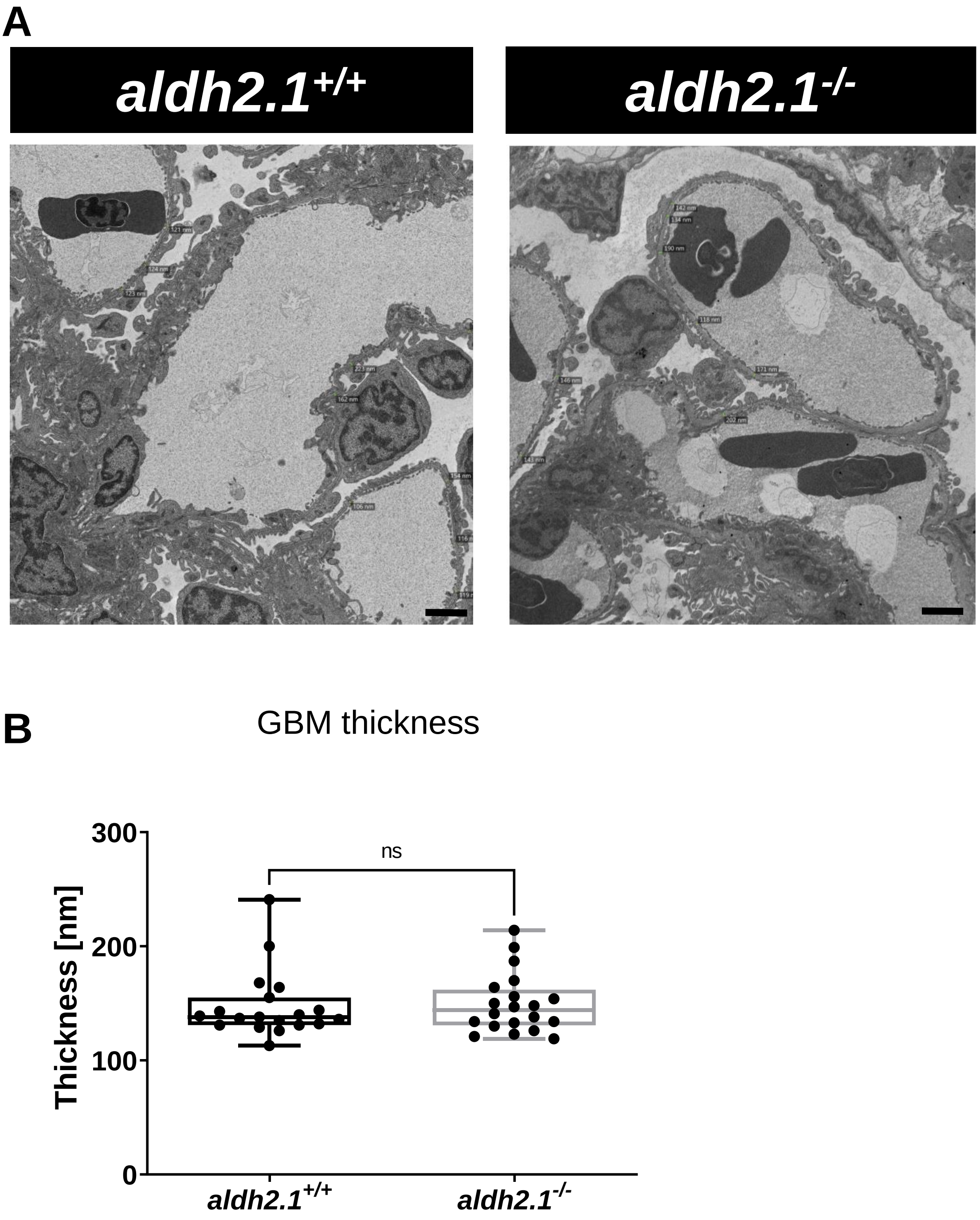

Figure S4. Unaltered glomerular morphology in adult *aldh2.1$^{-/-}$* zebrafish. A: Representative electronmicroscopy images showed no alterations in *aldh2.1$^{-/-}$* glomeruli. Black scale bar: 2 µm. **B:** Quantification of GBM thickness in *aldh2.1$^{-/-}$* zebrafish glomeruli showed no changes. Statistical analysis was done via Student's t-test, ns = not significant.

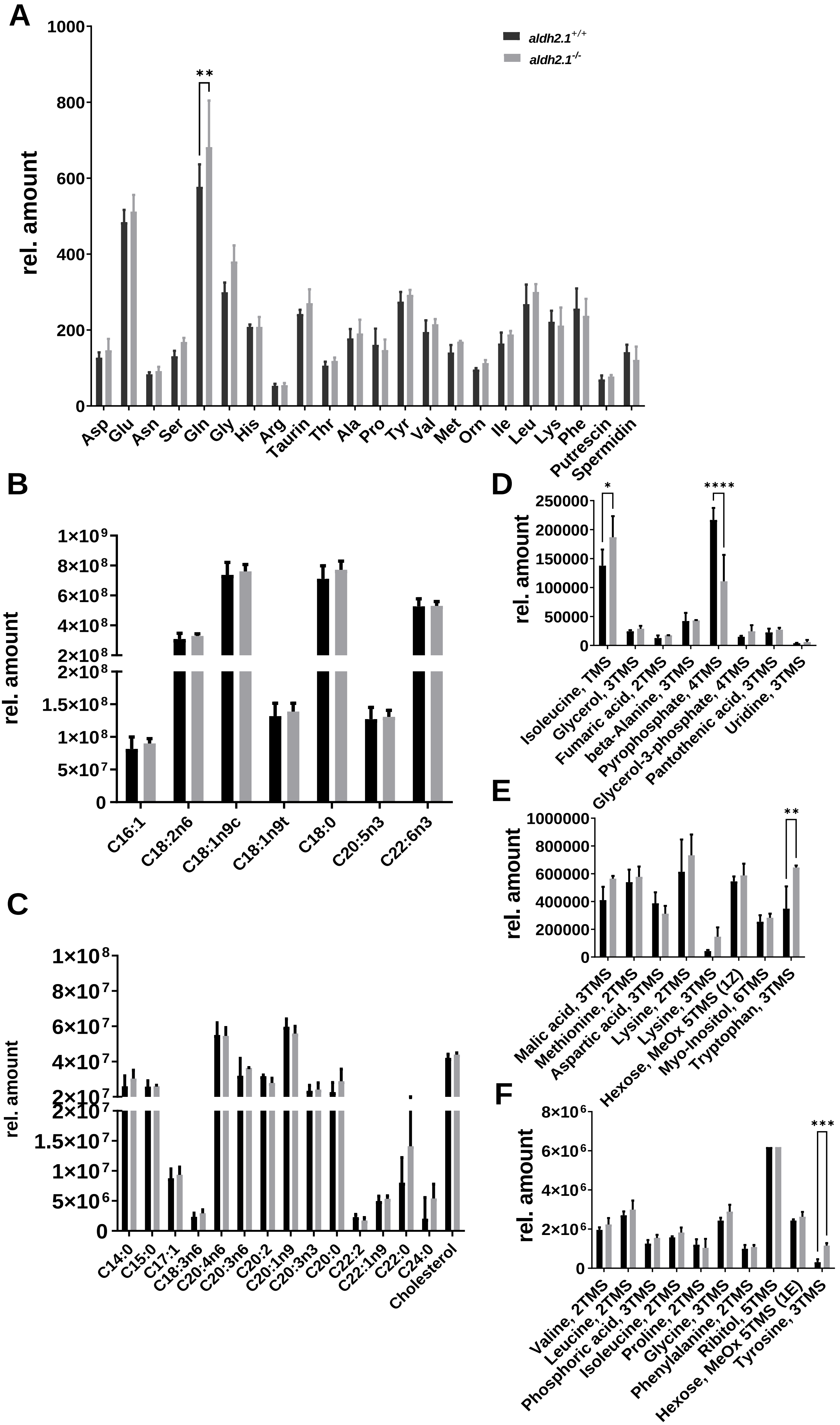
A
rel. amount
aldh2.1+/+
aldh2.1-/-
**
Asp Glu Asn Ser Gln Gly His Arg Taurin Thr Ala Pro Tyr Val Met Orn Ile Leu Lys Phe Putrescin Spermidin
B
rel. amount
C16:1 C18:2n6 C18:1n9c C18:1n9t C18:0 C20:5n3 C22:6n3
C
rel. amount
C14:0 C15:0 C17:1 C18:3n6 C20:4n6 C20:3n6 C20:2 C20:1n9 C20:3n3 C20:0 C22:2 C22:1n9 C22:0 C24:0 Cholesterol
D
rel. amount
*
****
Isoleucine, TMS
Glycerol, 3TMS
Fumaric acid, 2TMS
beta-Alanine, 3TMS
Pyrophosphate, 4TMS
Glycerol-3-phosphate, 4TMS
Pantothenic acid, 3TMS
Uridine, 3TMS
E
rel. amount
**
Malic acid, 3TMS
Methionine, 2TMS
Aspartic acid, 3TMS
Lysine, 2TMS
Lysine, 3TMS
Hexose, MeOx 5TMS (1Z)
Myo-Inositol, 6TMS
Tryptophan, 3TMS
F
rel. amount
***
Valine, 2TMS
Leucine, 2TMS
Phosphoric acid, 3TMS
Isoleucine, 2TMS
Proline, 2TMS
Glycine, 3TMS
Phenylalanine, 2TMS
Ribitol, 5TMS
Hexose, MeOx 5TMS (1E)
Tyrosine, 3TMS

Figure S5. Metabolomic screening showed minor alterations between *aldh2.1*$^{+/+}$ *and aldh2.1*$^{-/-}$ zebrafish larvae at 96 hpf. A: Quantification of amino acids displayed a significant increase in glutamine concentrations of *aldh2.1*$^{-/-}$ larvae*.* **B,C*:*** Quantification of fatty acids in 96 hpf larvae showed no change between *aldh2.1*$^{+/+}$ *and aldh2.1*$^{-/-}$ larvae. **D,E,F:** Semi-Targeted Screening identified an increase in aromatic amino acids: tryptophan and tyrosine and a decrease of pyrophosphates in *aldh2.1*$^{-/-}$ larvae. Statistical analysis was done via Student's t-test, ns = not significant, *p < 0.05, **p < 0.01, ***p < 0.001, ****p < 0.0001.

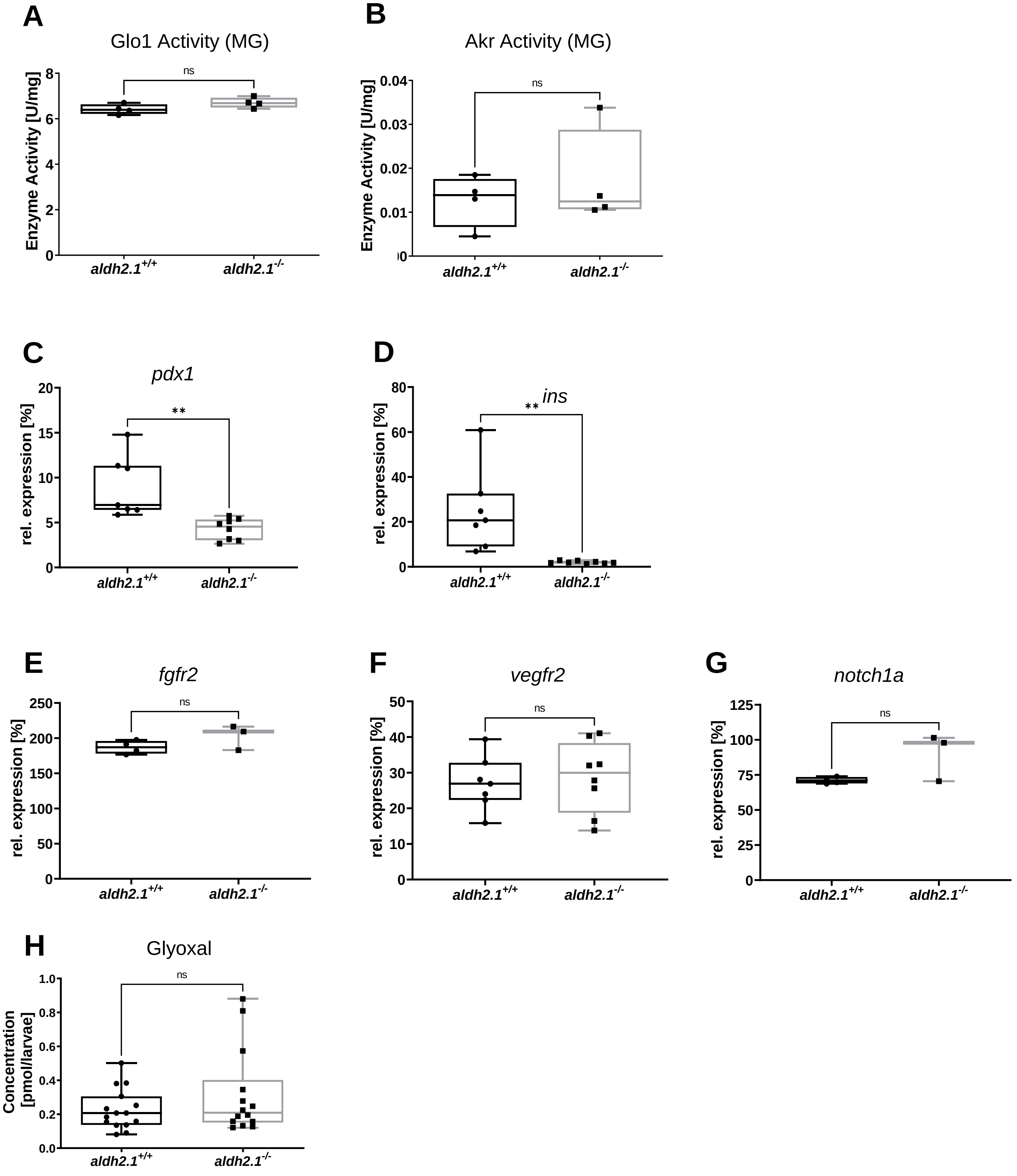

Figure S6. *aldh2.1$^{-/-}$* larvae displayed decreased *pdx1* and *ins expression,* but unaltered expression of angiogenesis related factors. A,B: Quantification of enzyme activity for Glo1 and Akr showed no change in *aldh2.1$^{-/-}$* zebrafish larvae. **C,D:** Strongly reduced *pdx1* and *ins* expression in *aldh2.1$^{-/-}$* larvae. **E,F,G:** Quantification of gene expression of angiogenesis related factors: *fgfr2* (**E**), *vegfr2* (**F**) and notch1a (**G**) showed no change in *aldh2.1$^{-/-}$* larvae compared to *aldh2.1$^{+/+}$* larvae. Expression was quantified via RT-qPCR with 96 hpf zebrafish larvae and normalized to arnt2, n = 5-8 clutches, 50 larvae per clutch. Quantification of glyoxal in 96 hpf larvae (**H**) showed no difference between *aldh2.1$^{+/+}$* and *aldh2.1$^{-/-}$* zebrafish, n = 14, 50 larvae per clutch. Statistical analysis was done via Student's t-test, ns = not significant, *p < 0.05, **p < 0.01, ***p < 0.001, ****p < 0.0001.

Name	NES	pvalue
Pantothenate_and_CoA_biosynthesis	-2,44	0,0002
Histidine_metabolism	-2,26	0,0006
Beta-Alanine_metabolism	-2,16	0,0007
Glycerolipid_metabolism	-2,1	0,0002
Ribosome	-2,08	0,0002
Pyruvate_metabolism	-2,03	0,0007
Valine,_leucine_and_isoleucine_degradation	-2	0,0009
Tryptophan_metabolism	-2	0,0004
Arginine_and_proline_metabolism	-1,99	0,0007
Lysine_degradation	-1,97	0,0007
Fatty_acid_degradation	-1,89	0,0018
Ascorbate_and_aldarate_metabolism	-1,87	0,0039
Glycolysis_Gluconeogenesis	-1,82	0,0015
VEGF_signaling_pathway	1,46	0,0245
Endocytosis	1,53	0,0003
Apoptosis	1,53	0,0028
MAPK_Signaling_Pathway	1,58	0,0003
Cardiac_muscle_contraction	1,58	0,0027
Cellular_senescence	1,6	0,0011
Ferroptosis	1,66	0,0066
Regulation_of_actin_cytoskeleton	1,73	0,0002
Tight_junction	1,88	0,0002
Focal_adhesion	1,99	0,0002
ECM-receptor_interaction	2,04	0,0002

Table S1. RNA-seq KEGG-Analysis values corresponding to Fig.4

CRISPR-construct name	Primer sequence (5‘ to 3‘)
Aldh2.1-CRISPR-for	TAGGGCTCTCCCTGGCGCCGCA
Aldh2.1-CRISPR-rev	AAACTGCGGCGCCAGGGAGAGC
**Genotyping primer name**	**Primer sequence (5‘ to 3‘)**
Aldh2.1- gt 2.1 fw1	CCCTGCTGTGCAGTGTATTG
Aldh2.1- gt 2.3 fw2	CTGGCATGGCAATAAACACA
Aldh2.1- gt 2.4 rv2	TGCATCGAGAGAGATGCTGCCT
**qPCR primer name**	**Primer sequence (5‘ to 3‘)**
aldh2.1-qPCR-left	CGCACTGTATATCGCCAGTTTA
aldh2.1-qPCR-right	GGACCAAACCCTGGGATAAT

Table S2. CRISPR constructs, primers for genotyping and qPCR for zebrafish *aldh2.1*

qPCR primer name	Primer sequence (5‘ to 3‘)	qPCR primer name	Primer sequence (5‘ to 3‘)
qPCR mapk3 fw2	GCTCCTGAGAGGGACAGTCATC	G6Pase_qPCR_Left	TCACAGCGTTGCTTTCAATC
qPCR mapk3 rv2	TCGCAGGTCGTCTGGAGTTTT	G6Pase_qPCR_Right	AACCCAGAAACATCCACAGC
qPCR mapk7 fw2	CTGGATCGGCCTTGTCGGT	G6PDH_qPCR_Left	CGTCTTTTGTGGCAGTCAGA
qPCR mapk7 rv2	GATCAATGGTTTCCGGCTGGC	G6PDH_qPCR_Right	TGATGGGTGGTGTTTTCTCA
qPCR mapk8 fw1	CCGGCCTCGCTAGCACA	cPEPCK_qPCR_Left	ATCACGCATCGCTAAAGAGG
qPCR mapk8 rv1	ACCTCGGTGGACATGGACGA	cPEPCK_qPCR_Right	CCGCTGCGAAATACTTCTTC
qPCR mapk11 fw1	GCTTCTTCGGGGCCTTAAGTACA	hk1_qPCR_Left	ATGATAGCGGCACAGCTTCT
qPCR mapk11 rv1	CATTCACGGCTACATTGCTTGGC	hk1_qPCR_Right	GTTGGTGTCTCGTGCCAATC
qPCR mapk12a fw2	ACCAAATGTTAACCCGCAAGCGAT	hk2_qPCR_Left	TGAGGTCAGTCTCGTCCAGT
qPCR mapk12a rv2	CCTCTGCTGCTGTTATCCGGC	hk2_qPCR_Right	TCTTAATCGACAGGCCACCG
qPCR mapk14b fw1	TCCCGGCACAGATCACATTG	gck_qPCR_Left	AATCACCGCTGACCTGCTAT
qPCR mapk14b rv1	TTTTCATCAAGAGCTCAGGCCC	gck_qPCR_Right	GCCACTTCACATACGCAATG
b2m_qPCR_Left	ACTGCTGAAGAACGGACAGG	glo1-qPCR-for	AGCAGACAATGCTGCGGGTG
b2m_qPCR_Right	GCAACGCTCTTTGTGAGGTG	glo1-qPCR-rev	CTACGGGAGAACGTCCAGGC
arnt2_qPCR_Left	AGCCAGACAGAGGTCTTCCA	2-INS_qPCR_Left	GGTCGTGTCCAGTGTAAGCA
arnt2_qPCR_Right	CCGAGGTCAGCAAAGTCTTC	2-INS_qPCR_Right	GGAAGGAAACCCAGAAGGGG
VEGFR2 (KDR):f1	TAAACAGCAGCGGTGTGCCA	Pdx1_RT-PCR_Left	ACACGCACGCATGGAAAGGACA
VEGFR2 (KDR):r1	CAGTCCACGTGGCCATCCATT	Pdx1_RT-PCR_Right	GCGGGCGCGAGATGTATTTGTT
FGFR2 F1	GGCCCATGAGCTCCCTGTTT	notch1a-qPCR-left	CATCACCCTTCCAGCAGTCT
FGFR2 R1	ACGGTCGGTCACTCACTGGA	notch1a-qPCR-right	CTGAAGAGCTCCACCCATGT

Table S3. qPCR primers
